# Intraoperative Blood Flow Analysis of DIEP vs. ms-TRAM Flap Breast Reconstruction Combining Transit-Time Flowmetry and Microvascular Indocyanine Green Angiography

**DOI:** 10.3390/jpm12030482

**Published:** 2022-03-16

**Authors:** Alexander Geierlehner, Raymund E. Horch, Ingo Ludolph, Andreas Arkudas

**Affiliations:** Department of Plastic and Hand Surgery and Laboratory for Tissue Engineering and Regenerative Medicine, University Hospital Erlangen, Friedrich Alexander University Erlangen-Nürnberg (FAU), 91054 Erlangen, Germany; raymund.horch@uk-erlangen.de (R.E.H.); ingo.ludolph@uk-erlangen.de (I.L.); andreas.arkudas@uk-erlangen.de (A.A.)

**Keywords:** microsurgery, flap imaging, perforator flaps, autologous breast reconstruction, free tissue transfer, indocyanine green angiography, transit-time flowmetry

## Abstract

Background: Vascular patency is the key element for high flap survival rates. The purpose of this study was to assess and compare the blood flow characteristics of deep inferior epigastric perforator (DIEP) and muscle-sparing transverse rectus abdominis musculocutaneous (ms-TRAM) flaps for autologous breast reconstruction. Methods: This prospective clinical study combined Transit-Time Flowmetry and microvascular Indocyanine Green Angiography for the measurement of blood flow volume, vascular resistance, and intrinsic transit time. Results: Twenty female patients (mean age, 52 years) received 24 free flaps (14 DIEP and 10 ms-TRAM flaps). The mean arterial blood flow of the flap in situ was 7.2 ± 1.9 mL/min in DIEP flaps and 11.5 ± 4.8 mL/min in ms-TRAM flaps (*p* < 0.05). After anastomosis, the mean arterial blood flow was 9.7 ± 5.6 mL/min in DIEP flaps and 13.5 ± 4.2 mL/min in ms-TRAM flaps (*p* = 0.07). The arterial vascular resistance of DIEP flaps was significantly higher than that of ms-TRAM flaps. The intrinsic transit time of DIEP flaps was 52 ± 18 s, and that of ms-TRAM flaps was 33 ± 11 s (*p* < 0.05). The flap survival rate was 100%. One DIEP flap with the highest intrinsic transit time (77 s) required surgical revision due to arterial thrombosis. Conclusion: In this study, we established the blood flow characteristics of free DIEP and ms-TRAM flaps showing different blood flow rates, vascular resistances, and intrinsic transit times. These standard values will help to determine the predictive values for vascular compromise, hence improving the safety of autologous breast reconstruction procedures.

## 1. Introduction

Nowadays, abdominal tissue as the main source for breast reconstruction is preferably harvested either as a complete muscle-preserving deep inferior epigastric perforator (DIEP) flap or as a muscle-sparing transverse rectus abdominis musculocutaneous (ms-TRAM) flap [1,2]. The ability to reconstruct the female breast in a like-with-like fashion with low donor site morbidity has led to its widespread use [3]. Some of the latest tissue engineering and regenerative medicine methods aiming to overcome donor site sequelae are promising but not yet clinically feasible [4,5]. Although both DIEP and ms-TRAM flaps have overall low complication rates, partial flap necrosis and total flap loss due to vascular compromise remain imminent postoperative risks [6]. Sufficient vascular perfusion remains a key aspect for the overall outcome and flap survival. In the last few years, numerous clinical studies assessed the intra- and postoperative perfusion of free flaps using different technologies [7,8,9,10,11,12,13,14]. However, several studies aiming to understand the hemodynamics of free flaps showed methodological flaws such as a heterogeneous study population, small sample sizes of the included flap types in terms of tissue composition, the sole assessment of arterial flow characteristics, or the use of nowadays outdated technologies [15,16,17,18,19]. This study measures and compares intraoperative arterial and venous blood flow and perfusion characteristics of DIEP and ms-TRAM flaps for breast reconstruction combining Transit-Time Flowmetry (TTFM) and Indocyanine Green Angiography at a microscopic level (mICG-A). TTFM, an ultrasound-based technology for the assessment of vascular blood flow, was originally introduced into clinical practice for cardiac surgery [20,21]. Validation studies showed highly accurate and reproducible measurements which enabled its extension towards other surgical specialties such as vascular surgery and microsurgery [22,23,24]. The intravenous application of Indocyanine Green in combination with microscope-integrated fluorescence-based video angiography (IR800) and the analysis tool FLOW800 (Carl Zeiss, Oberkochen, Germany) enables the recording, measurement, and assessment of the microvascular patency and blood flow characteristics of vessels just a few millimeters in diameter [25,26]. The assembly of these two state-of-the art technologies is considered a novel approach. We believe that an advanced understanding of their hemodynamic properties will improve the safety of the two most commonly used free flaps for autologous breast reconstruction. This study further aimed to establish normative blood flow and perfusion values as groundwork for the determination of predictive values for postoperative thrombotic events.

## 2. Materials and Methods

Patients receiving DIEP or ms-TRAM flaps for breast reconstruction were enrolled in this prospective mono-centered clinical study. The study was approved by the Ethical Committee in accordance with the Declaration of Helsinki. Prior to study inclusion, written consent was given by each patient.

### 2.1. Surgical Technique

Autologous breast reconstructions were performed by three experienced surgeons. All patients received a standardized computed tomography angiography (CTA) of the abdomen for perforator mapping prior to surgery. Depending on the anatomy of the selected perforators (size, course, and number), the patients included in this study received DIEP or ms-TRAM free flaps for autologous breast reconstruction. Our postoperative anticoagulation regimen usually consists of low-molecular-weight heparin application subcutaneously until full mobilization is achieved. If contraindications for low-molecular-weight heparin exist, patients usually receive weight-adjusted unfractionated heparin. Patients suffering from hyperthyroidism, thyroid adenoma or autonomy, and known allergies/hypersensitivity to Indocyanine green or sodium iodide were excluded. The patient body temperature was kept stable by using a warming mattress (37 °C) and maintaining the ambient temperature between 20 °C and 22 °C. All patients received a balanced intraoperative crystalloid volume substitution of, on average, 53 mL/kg (mean: 3780 ± 1710 mL) in order to maintain stable hemodynamic conditions. The average intraoperative blood loss was 140 ± 100 mL. The internal mammary artery (IMA) and vein (IMV) were used as recipient vessels in all cases. Each flap was harvested in a standardized fashion with the inferior epigastric artery and the inferior epigastric vein as vascular pedicle dissected towards their origin at the external iliac artery and vein. Each vascular pedicle consisted of one artery and one vein. DIEP and ms-TRAM flaps with more than one venous anastomosis were excluded from this study. All arterial and venous anastomoses were performed end to end. Arterial anastomoses were hand-sewn with interrupted nylon sutures, whereas all venous anastomoses were performed using a venous coupler device (Synovis Micro Companies Alliance, Inc., Birmingham, AL, USA).

### 2.2. Transit-Time Flow Measurement (TTFM)

**Figure 1:**
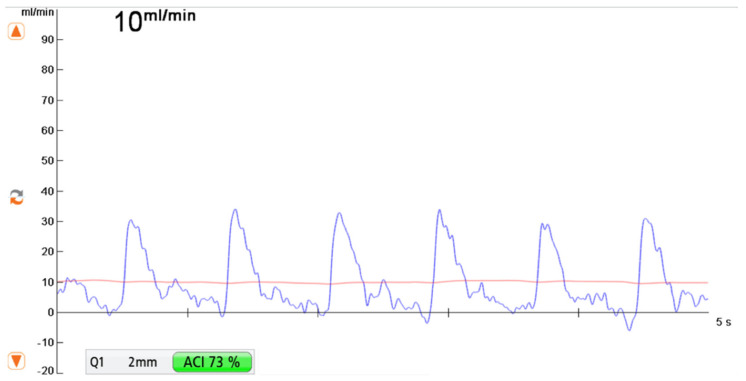
Transit-Time Flow Volume Measurement (TTFM) showing a flow volume of 10 mL/min with an Acoustic Coupling Index (ACI) of 73% using a 2 mm probe.

MiraQ™ Vascular (Medistim ASA, Oslo, Norway) was used for intraoperative blood flow measurements. The probe diameter ranged from 1.5 to 4 mm depending on the vessel size. Blood flow values were recorded for several minutes until a steady curve of blood flow occurred (Figure 1). Arterial and venous blood flow volume measurements were performed at three predefined time points during surgery:

- Measurement F was taken after flap elevation and isolation on its vascular pedicle prior to free flap transfer
- Measurement R was performed at the recipient vessel prior to anastomosis.
- Measurement AA was taken at the vascular pedicle after anastomosis and flap reperfusion.

The mean arterial pressure was measured and documented at each measurement time point.

### 2.3. Microvascular Indocyanine Green Angiography (mICG-A)

**Figure 2:**
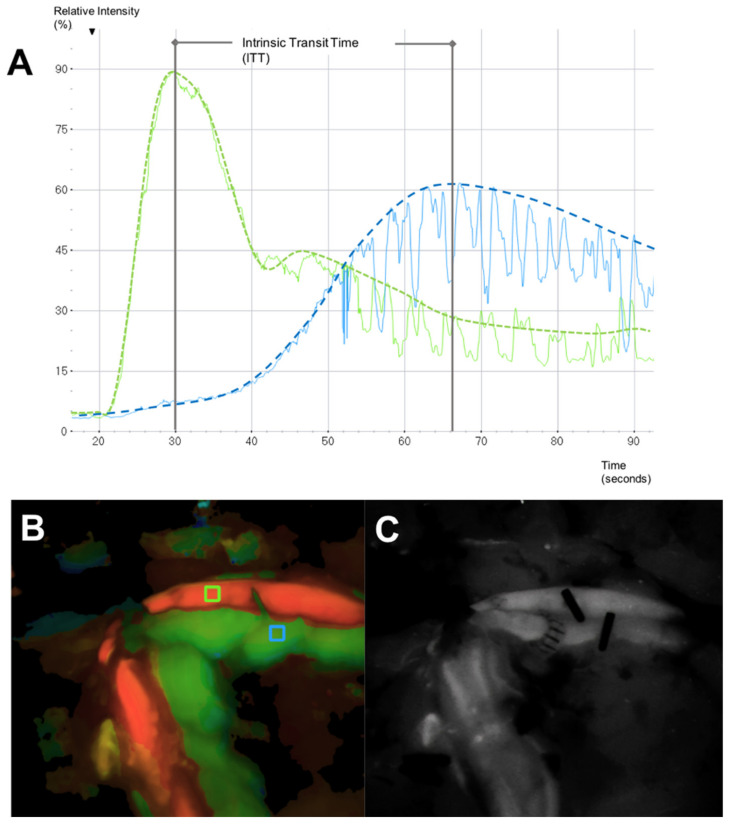
(**A**) Microvascular Indocyanine Green Angiography (mICG-A) flow curves in two selected regions of interest (ROI) (green curve: arterial flow, blue curve: venous flow). The spikes are artefacts caused by respiratory movements. (**B**) Delay Map obtained with FLOW800 illustrating both ROIs (green ROI placed at the artery, blue ROI placed at the vein) and picturing the two flow curves. (**C**) Gray-scale map of fluorescence intensity (Intensity Map) illustrating both artery and vein after anastomosis.

In this clinical study, Indocyanine Green (ICG) was administered as an intravenous bolus (3 mL VERDYE 5 mg/mL) after arterial and venous anastomosis and flap reperfusion. The anastomosed flap pedicle was placed below the microscope (KINEVO 900, Carl Zeiss, Oberkochen, Germany). Recordings of the supplying artery and draining vein started immediately after intravenous ICG application and were continued until the intensity of the ICG markedly decreased in the artery and vein. Intraoperative fluorescence analysis requires the selection of certain regions of interest (ROI). Two ROIs were placed at the flap pedicle, one at the supplying artery, and the other at the draining vein close to the anastomosis, uncovered from any surrounding tissue (Figure 2). FLOW800 measures the intensity of ICG in the regions of interest for a time period and enables the instant visualization of blood flow variations within small vessels. The time between the maximum ICG intensity of arterial inflow and venous outflow is defined as Intrinsic Transit Time (ITT), which is considered as a parameter of blood flow velocity within the flap (Figure 2) [27].

### 2.4. Vascular Resistance

Arterial vascular Resistance (*aVR*) was calculated as millimeters of mercury per milliliter per minute (mmHg/mL/min) based on a previously reported method in adherence to the Poiseuille’s Law, using the following formula: [28,29]

R=ΔP/Q

*R* = *aVR* = arterial vascular resistance, Δ*P* = blood pressure gradient = mean arterial blood pressure (*MAP*)–mean venous blood pressure (MVP). The mean venous blood pressure (MVP) is estimated to be close to 0 and, as a result, disregarded in the calculation of vascular resistances. This results in the following formula: [28,29,30,31]

aVR=MAP/aBF

### 2.5. Data Analysis and Synthesis

Descriptive analysis was performed for patient demographics. Data are shown as mean ± standard deviation. Changes in blood flow and vascular resistance between the different time points within one group were calculated using the paired Student’s *t*-test. Blood flow and vascular resistance between DIEP and ms-TRAM flaps at different time points was analyzed using an unpaired Student’s *t*-test. Nonparametric data were analyzed with the Wilcoxon matched-pairs rank test within one group, whereas the Mann–Whitney U test was used for analyses of nonparametric data between DIEP and ms-TRAM flaps. The correlation of data assuming Gaussian distribution was calculated using the Pearson correlation coefficient. The Spearman’s rank correlation coefficient was used for data not passing a test for normality. The significance level was set at *p* < 0.05. Three outliers (one arterial and two venous blood flow values at the recipient site (Measurement *R*) before anastomosis) were identified using the ROUT method (*Q* = 1%) and appropriately excluded from statistical analysis. Statistical analyses and graphic illustrations were performed using GraphPad Prism (GraphPad Software, Inc., San Diego, CA, USA).

## 3. Results

A total of 20 female patients receiving 24 DIEP or ms-TRAM flaps for breast reconstruction were included in this prospective study. Patients’ average age was 52 years, ranging from 39 to 68 years. Fourteen flaps were harvested as DIEP (57%), and 10 as ms-TRAM (43%). Seven ms-TRAM flaps were classified as ms1-TRAM flap, and three as ms2-TRAM flap, according to the classification by Nahabedian et al. (Table 1) [32]. The median flap weight was 435 g, ranging from 299 to 1169 g. The median weight of DIEP flaps (390 g) was not significantly different from that of ms-TRAM flaps (491 g). The average flap ischemia time was 46 min. The venous coupler size ranged from 2.5 to 3.5 mm.

### 3.1. Blood Flow Volume (mL/min)

**Figure 3:**
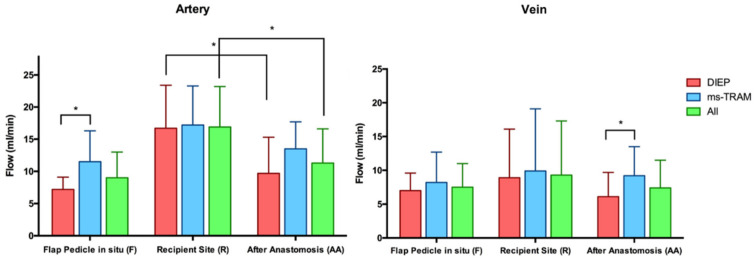
Arterial and venous blood flows (mL/min) at three predefined time points (F, R, AA). The bars represent means ± standard error (* indicates significant differences).

The average blood flow of the flap artery isolated as pedicle prior to free tissue transfer (F) was 9 ± 4 mL/min (mean ± SD). Its venous outflow was lower (7.5 ± 3.5 mL/min), resulting in an artery-to-vein (A/V) flow ratio of 1.4 ± 0.7. The mean blood flow of the recipient internal mammary artery and vein prior to flap anastomosis (R) was 16.9 ± 6.3 and 9.4 ± 8 mL/min, respectively. After anastomosis (AA), the arterial and venous blood flow volume was 11.3 ± 5.3 and 7.4 ± 4.1 mL/min, respectively, with an A/V flow ratio of 1.8 ± 1.3. The arterial blood flow of the intact recipient artery (R) significantly decreased after anastomosis with the flap artery (AA) (*p* = 0.002). However, the arterial and venous blood flow of the included flaps did not significantly change after flap transfer. (Figure 3) The blood flow of the intact recipient artery (R) did not alter the blood flow of the flaps after anastomosis. There was a significant positive correlation between the arterial inflow and the venous outflow both before (F) and after anastomosis (AA) (*p* < 0.05). The arterial and venous blood flow rates before and after anastomosis in DIEP flaps were lower than in ms-TRAM flaps (Table 2). There was no correlation between the arterial blood flow volume and the flap weight. The flap ischemia time did not change the blood flow rates of the examined flaps.

### 3.2. Vascular Resistance (mmHg/mL/min)

**Figure 4:**
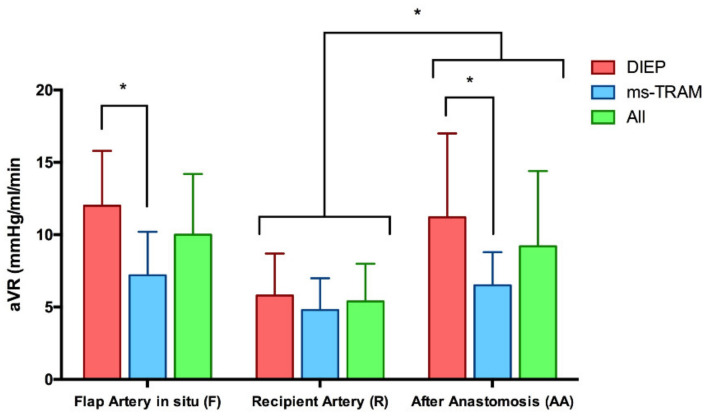
Arterial Vascular Resistance (aVR) at three predefined time points (F, R, AA). The bars represent means ± standard error (* indicates significant differences).

The mean arterial vascular resistance (aVR) of the included flaps prior to tissue transfer (10 ± 4.2 mmHg/mL/min) did not significantly change after anastomosis (9.2 ± 5.2 mmHg/mL/min). The vascular resistance of the recipient artery, however, significantly increased from 5.4 ± 2.6 to 9.2 ± 5.2 mmHg/mL/min after anastomosis to the flap (*p* < 0.001). The average arterial vascular resistance (aVR) of DIEP flaps prior to (F) and after flap transfer (AA) was 12 ± 3.8 and 11.2 ± 5.8 mmHg/mL/min, respectively. By contrast, ms-TRAM flaps had significantly lower arterial vascular resistance values prior to (7.2 ± 3 mmHg/mL/min; *p* = 0.004) and after flap reperfusion (6.5 ± 2.3 mmHg/mL/min; *p* = 0.02) (Figure 4 and Table 3). There was no correlation between the arterial vascular resistance and the weight of the included flaps before or after flap transfer.

### 3.3. Intrinsic Transit Time

**Figure 5:**
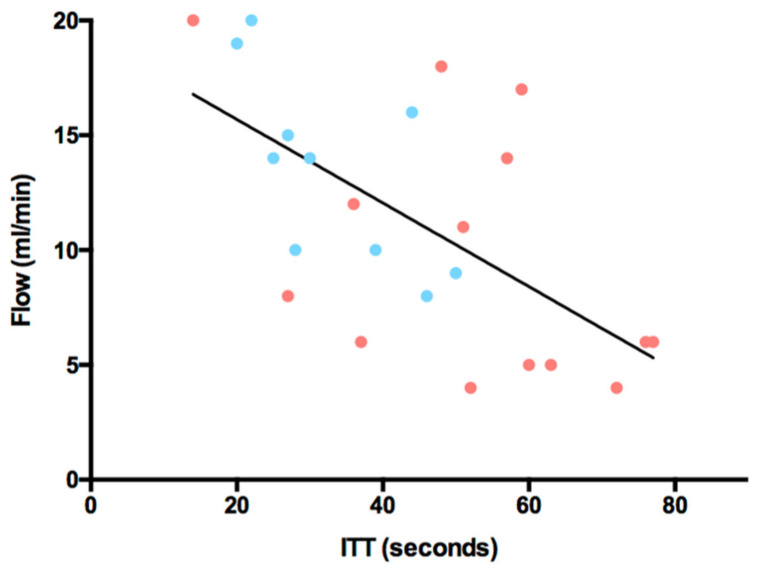
Arterial blood flow (ml/min) versus Intrinsic Transit Time (ITT, seconds); y = −0.182x + 19.33; *p* = 0.001; r2 = 0.3875; red dots = DIEP flaps; blue dots = ms-TRAM flaps.

**Figure 6:**
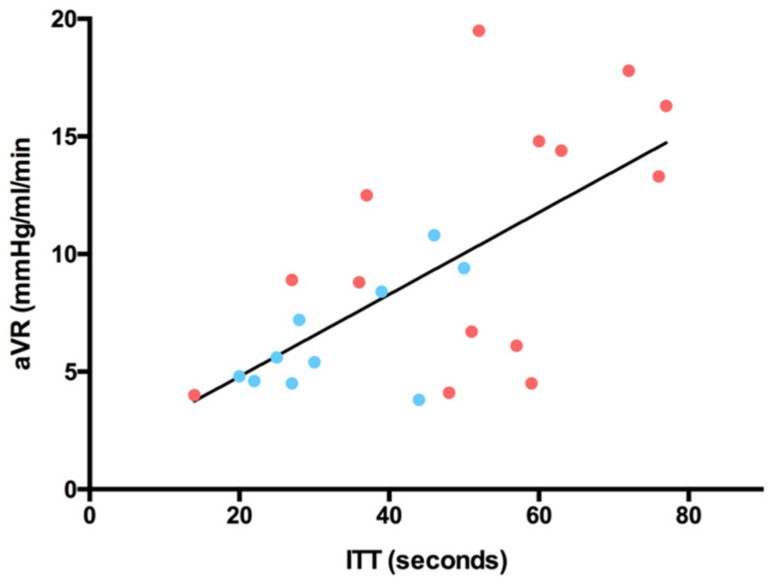
Arterial Vascular Resistance (aVR, mmHg/mL/min) versus Intrinsic Transit Time (ITT, seconds); y = 0.1744x + 1.308; *p* = 0.0006; r2 = 0.4196; red dots = DIEP flaps; blue dots = ms-TRAM flaps.

The mean Intrinsic Transit Time (ITT) after flap reperfusion was 44 ± 18 s (s), ranging from 14 to 77 s. The average ITT of DIEP flaps (52 ± 18 s) was significantly higher than the average ITT of ms-TRAM flaps (33 ± 11 s) (*p* = 0.005). The average vascular resistance at the time of ITT measurements was 9 ± 4.7 mmHg/mL/min. There was a significant negative correlation between the arterial blood flow and the ITT after anastomosis (*p* = 0.001) (Figure 5). By contrast, a significant positive correlation was seen between the arterial vascular resistance (aVR) and the ITT after anastomosis (*p* = 0.0006) (Figure 6). There was no correlation between the ITT and flap ischemia time, flap weight, or mean arterial pressure (MAP).

Of all included free flaps, one DIEP flap required surgical revision due to a thrombotic event occurring on the fourth day after autologous breast reconstruction. The ITT of this flap was 77 s. After emergency thrombectomy, no further complication occurred. The overall flap survival rate was 100%.

## 4. Discussion

Numerous recently developed technologies enable the illustration and measurement of vascularity and perfusion in free flaps at a pre-, intra, or postoperative stage, with the ultimate goal to increase their safety and efficacy [7,11,12,18,33,34,35,36]. The combination of Transit-Time Flowmetry (TTFM) and microvascular Indocyanine Green Angiography (mICG-A) is considered a unique approach aiming to meticulously evaluate and compare the intraoperative blood flow characteristics of DIEP and ms-TRAM flaps. A recent study successfully established this combination for the detection of early venous congestion in an animal flap model [37]. However, no study so far assessed the combined potential of these techniques in a clinical setting for autologous breast reconstructions. Our results show that the overall arterial blood flow of both DIEP and ms-TRAM flaps did not significantly increase after anastomosis with the recipient internal mammary vessel. The blood flow of the intact recipient artery did not influence the arterial blood flow of the included flaps. In fact, it seemed to be the opposite. In this study, both DIEP and ms-TRAM flaps downregulated the recipient artery flow towards blood flow values of the in situ flap prior to tissue transfer. These observations are supported by other studies showing that the flow of the recipient artery can either be down- or upregulated after flap anastomosis, approximating blood flow values of the flap isolated on its pedicle before tissue transfer [19,38,39,40]. Lorenzetti et al. measured the blood flow of the thoracodorsal artery before and after anastomosis with ms-TRAM flaps and reported an upregulation of the recipient artery. Before anastomosis, the thoracodorsal artery had relatively low blood flow values (4.9 ± 3 mL/min) in situ. However, after anastomosis with the ms-TRAM flap, the blood flow increased (13.7 ± 5 mL/min) towards the original blood flow rate of the isolated flap pedicle in situ before tissue transfer [38]. This phenomenon was observed not only in fasciocutaneous but also in musculocutaneous and muscle free flaps and therefore seems to be irrespective of the tissue composition [40]. Previous studies reported generally different blood flow rates and vascular resistances in fasciocutaneous, musculocutaneous, muscle, and intraperitoneal flaps [31,38]. These findings support the notion that both blood flow and vascular resistance depend on the type of tissue and its relative proportion. The tissue composition determines the vascularity of each flap, which at the same time, reflects the vascular resistance. Free flaps mainly composed of muscle tissue contain a rich vascular network connected by resistance vessels, resulting in a lower vascular resistance than fasciocutaneous flaps with a rather sparse network of much smaller vessels [30,31]. In our study, ms-TRAM flaps had an average arterial blood flow of 13.5 mL/min and a vascular resistance of 6.4 mmHg/mL/min after anastomosis. By contrast, DIEP flaps showed significantly lower blood flow values and consequently a higher vascular resistance. Although both flaps were, apart from a small segment of the rectus abdominis muscle in ms-TRAM flaps, grossly composed of the same tissue, the difference in vascular resistance seemed to be a matter of vascularity. We theorize that a larger number of perforators in ms-TRAM flaps was the main reason for a lower vascular resistance, hence providing a higher overall and weight-adjusted arterial blood flow in comparison to DIEP flaps. The overall arterial inflow of the included flaps was about 1.4 to 1.8 times greater than the venous outflow. Selber et al. reported similar results in ms-TRAM and other fasciocutaneous flaps [41]. We theorize that the peripheral leakage of small blood vessels at the flap edges caused the disparity between arterial inflow and venous outflow. An A/V ratio of more than 1 seems to a certain level inevitable and needs to be considered by surgeons during free flap flow measurements.

Microvascular Indocyanine Green Angiography (mICG-A) combined with the microscope-integrated software FLOW800 provides valuable information on vascular patency and enables the real-time visualization of arterial in- and venous outflow in free flaps [42,43,44]. It is a matter of common pathophysiological knowledge that the alteration in blood flow, as part of the Virchow’s triad, is a main contributor to thrombus formation in blood vessels [45,46]. Previous studies have already theorized that a prolonged ITT might be an indicator of low blood flow velocities, hence accounting for increased vascular resistances [27]. The combination of TTFM with mICG-A enabled to measure and detect a positive correlation of ITT with vascular resistance in free flaps. ITT, similar to blood flow, seems to depend on the flap tissue composition. In our study, the DIEP flap, which was composed of fasciocutaneous tissue, had a significantly higher average ITT (52 s) than the ms-TRAM flap (33 s), classified as musculocutaneous flap. These observations are supported by a previous study measuring a shorter ITT in muscle flaps (27.7 s) than in fasciocutaneous flaps (47.5 s) [26]. Holm et al. reported that an ITT of more than 50 s was associated with an increased risk for vascular compromise and surgical revision in free tissue transfer procedures [27]. The study, however, showed essential methodological flaws such as a heterogenous study population with varying free flap entities. In our study, eight flaps surpassed the threshold of 50 s without any hemodynamic postoperative complication. Only one DIEP flap with an ITT of 77 s required surgical revision due to a thrombotic event several days after flap transplantation. Although this was the highest ITT of all included flaps, the scarce occurrence of just a single hemodynamic complication several days after autologous breast reconstruction did not allow drawing any correlation between a prolonged ITT and the increased risk of postoperative hemodynamic complications. To the best of our knowledge, this is the first study that measures, compares, and detects hemodynamic differences between DIEP and ms-TRAM flaps. The clinical relevance of this study is the establishment of standard values of intraoperative hemodynamic and perfusion properties of DIEP and ms-TRAM flaps. We could detect significant differences in hemodynamics properties between DIEP and ms-TRAM flaps. Flaps with abnormally high or low blood flow values, according to our newly established standard hemodynamic characteristics, made a closer intraoperative assessment of anastomotic patency necessary. We are aware that these techniques do not replace a clinical examination but rather help to improve our intraoperative decision-making process. The results of this study, however, do not provide any recommendation in terms of favoring one or the other free flap type for autologous breast reconstruction. Historically, the choice between DIEP and ms-TRAM flap has been a far more extensive topic that needs to take numerous other variables into account. Our meticulous assessment of arterial and venous blood flow, arterial vascular resistance, and ITT at crucial intraoperative time points enables the establishment of normative values. This should help to assess vascular patency especially in cases where the surgeon or devices such as a regular hand-held Doppler fail to detect a more subtle vascular compromise.

## 5. Conclusions

In this study, we evaluated the hemodynamic characteristics of free DIEP and ms-TRAM flaps. The combination of Transit-Time Flowmetry and microvascular Indocyanine Green Angiography enabled the qualitative and quantitative intraoperative assessment of anastomotic patency. Our study serves as fundamental work for the determination of predictive values for postoperative thrombotic events and of cut-off values that will ease intraoperative decision making in the future.

## Figures and Tables

**Table 1:** Characteristics of the included patients. Abbreviations: DIEP: deep inferior epigastric perforator; ms-TRAM: muscle-sparing transverse rectus abdominis musculocutaneous.

Patient Number	Age	Type of Flap	Flap Weight	Ischemia Time	Intrinsic Transit Time	Hemodynamic Postoperative Complications
	(Years)		(g)	(min)	(s)	
1	46	DIEP	459	70	37	-
2	55	ms-TRAM	340	49	46	-
		DIEP	314	53	36	-
3	57	DIEP	1036	45	27	-
4	46	ms-TRAM	344	46	50	-
5	39	ms-TRAM	1169	35	22	-
6	62	DIEP	309	37	60	-
7	49	DIEP	370	56	14	-
		ms-TRAM	310	64	25	-
8	47	DIEP	514	42	76	-
9	57	ms-TRAM	328	56	30	-
10	55	DIEP	591	54	59	-
11	51	DIEP	352	50	77	Arterial Thrombosis
12	55	ms-TRAM	464	42	28	-
		ms-TRAM	517	32	27	-
13	44	DIEP	365	44	72	-
14	56	DIEP	310	48	48	-
15	49	DIEP	688	44	63	-
16	43	DIEP	299	41	51	-
17	64	ms-TRAM	691	41	20	-
		ms-TRAM	810	40	44	-
18	68	DIEP	713	43	57	-
19	42	ms-TRAM	631	38	39	-
20	51	DIEP	410	42	52	-

**Table 2:** Comparison of the Blood flow (mL/min) of the Flap Pedicle in situ (F), of the Recipient Artery (R), and After Anastomosis (AA).

		Flow in mL/in (Mean ± SD)								*p*-Value		
		Flap Pedicle In Situ (F)			Recipient Vessel (R)		After Anastomosis (AA)					
Type of Flap	No. of Flaps	Artery	Vein	Ratio A/V	Artery	Vein	Artery	Vein	Ratio A/V	Artery vs. Vein	F vs. AA	R vs. AA
All	24	9 ± 4	7.5 ± 3.5	1.4 ± 0.7	16.9 ± 6.3	9.4 ± 8	11.3 ± 5.3	7.4 ± 4.1	1.8 ± 1.3	0.07 (F); **0.0001** (AA)	0.1 (A); 0.9 (V)	**0.002** (A); 0.4 (V)
DIEP	14	7.2 ± 1.9	7 ± 2.6	1.1 ± 0.3	16.7 ± 6.7	8.9 ± 7.2	9.7 ± 5.6	6.1 ± 3.6	1.9 ± 1.6	0.5 (F); **0.002** (AA)	0.2 (A); 0.5 (V)	**0.01** (A); 0.2 (V)
ms-TRAM	10	11.5 ± 4.8	8.2 ± 4.5	1.8 ± 1	17.2 ± 6.1	9.9 ± 9.2	13.5 ± 4.2	9.2 ± 4.3	1.7 ± 0.8	**0.04** (F); **0.02** (AA)	0.1 (A); 0.5 (V)	0.1 (A); 0.9 (V)
*p*-value (DIEP vs. ms-TRAM)		**0.02**	0.5	0.06	0.85	0.96	0.07	**0.04**	0.8			

Bold numbers indicate significant differences.

**Table 3:** Comparison of Arterial Vascular Resistances (mmHg/mL/min) of the flap pedicle in situ (F), of the Recipient Artery (R) and After Anastomosis (AA).

	Arterial Vascular Resistance (aVR) in mmHg/mL/min (Mean ± SD)			*p*-Value	
Type of Flap	Flap Artery (FA)	Recipient Artery (RA)	After Anastomosis (AA)	FA vs. AA	RA vs. AA
All	10 ± 4.2	5.4 ± 2.6	9.2 ± 5.2	0.5	**0.0002**
DIEP	12 ± 3.8	5.8 ± 2.9	11.2 ± 5.8	0.7	**0.005**
ms-TRAM	7.2 ± 3	4.8 ± 2.2	6.5 ± 2.3	0.5	**0.02**
*p*-value (DIEP vs. ms-TRAM)	**0.004**	0.4	**0.02**		

The bold numbers indicate significant differences.

## Data Availability

The datasets generated during the current study are available from the corresponding author on reasonable request.
